# How do midwives facilitate women to give birth during physiological second stage of labour? A protocol for a systematic review

**DOI:** 10.1186/s13643-018-0916-1

**Published:** 2019-01-03

**Authors:** Corine J. Verhoeven, Dale Spence, Viola Nyman, René H. J. Otten, Maria Healy

**Affiliations:** 1 Amsterdam UMC, Department of Midwifery Science, AVAG, the Amsterdam Public Health research institute, Vrije Universiteit Amsterdam, Van der Boechorststraat 7, Amsterdam, 1081 BT The Netherlands; 2 Department of Obstetrics and Gynaecology, Maxima Medical Centre, Veldhoven, Netherlands; 3 School of Nursing and Midwifery, Queen’s University Belfast, 97 Lisburn Road, Belfast, BT9 7BL Northern Ireland; 4 Department of Research and Development, NU-Hospital Group, Trollhattan, Sweden; 5 Institute of Health and Care Sciences, University of Gothenburg, Gothenburg, Sweden; 6 Amsterdam UMC, Vrije Universiteit Amsterdam, de Boelelaan 1117, Amsterdam, Netherlands

**Keywords:** Intrapartum care, Midwifery, Physiological birth, Second stage of labour

## Abstract

**Background:**

Midwives’ practices during the second stage of labour vary nationally and internationally. We aim to retrieve evidence that supports high-quality intrapartum care by conducting a systematic review of the literature.

**Methods:**

Electronic bibliographic databases including PubMed, EMBASE.com, Cumulative Index to Nursing and Allied Health Literature (CINAHL), PsycINFO, Maternity and Infant Care Database (through MIDIRS), and The Cochrane Library will be searched to identify studies that meet the inclusion criteria. No language or publication date constraints will be applied. Articles that pass the two-stage screening process will then be assessed for risk of bias and have their reference lists hand searched.

**Discussion:**

A midwife’s practice can be influenced by education and cultural practices but ultimately it should be informed by up-to-date research evidence. By analysing and synthesising the results of the studies, this systematic review will provide valuable insight into high-quality evidence-based midwifery care, which can inform practice, education and future research.

**Systematic review registration:**

PROSPERO CRD42018088300

**Electronic supplementary material:**

The online version of this article (10.1186/s13643-018-0916-1) contains supplementary material, which is available to authorized users.

## Background

Labour and birth constitute significant and memorable life events for a woman and her wider family. How a woman experiences birth has both short- and long-term effects on health and wellbeing for both herself and her baby [2, 4, 5, 9, 10, 14]. Experiencing a physiological labour and birth may contribute to positive outcomes: “The health and well-being of a mother and child at birth largely determines the future health and wellness of the entire family” [15].

As far back as 1997, the WHO defined physiological birth as spontaneous onset, low risk at the commencement of labour and continuing so for the remainder of labour and birth. The infant is born spontaneously, between 37 and 42 weeks of pregnancy with a cephalic presentation. Following birth, both mother and infant are in good condition (World Health Organization, 1997). Labour can be divided into three stages: the first, second and the third stage of labour. The WHO (2018) have recently defined the first stage of labour as the time period characterised by regular painful uterine contractions until full dilatation of the cervix and the second stage of labour as the time period between full dilatation of the cervix and the birth of the baby, whilst the woman is experiencing an involuntary urge to bear down, due to expulsive uterine contractions. The third stage is recognised as the period after the birth of the baby ending with the birth of the placenta and fetal membranes [1].

Midwives can facilitate the process of physiological labour and birth by enabling the interplay of reproductive hormonal and neuro-hormonal mechanisms by their kind and respectful caring practices, which promote oxytocin release for effective uterine contractions during labour and the relaxation of the birth canal [12, 13]. However, there is little explanation or description of the variety of physical and emotional actions the midwife does when “being with” a woman during birth of the baby, in particular, how they facilitate this physiological process. Furthermore, Kennedy et al. emphasised the priority of research that “identifies and describes aspects of care that optimise, and those that disturb, the biological/physiological processes for healthy childbearing women and fetus/newborn infants and those who experience complications” ([8] p e777).

Therefore, we plan to undertake a systematic review to identify pertinent evidence related to intrapartum midwifery care, focusing specifically on care during the second stage of labour.

This leads to our structured research questions which were formulated using the PICO (Patient or Population, Intervention, Comparison, Outcome) framework for quantitative research and the PEO (Population, Exposure, Outcomes) question format for qualitative research questions: “How do midwives facilitate women to give birth during physiological second stage of labour?” and “What evidence supports good quality intrapartum care during the second stage of labour?”

The aim of the systematic review is to collate, analyse and synthesise the international evidence that supports high-quality intrapartum care during the second stage, which will inform midwifery practice, education and future research and positively influence this aspect of midwifery care for women.

## Methods

**Fig. 1:**
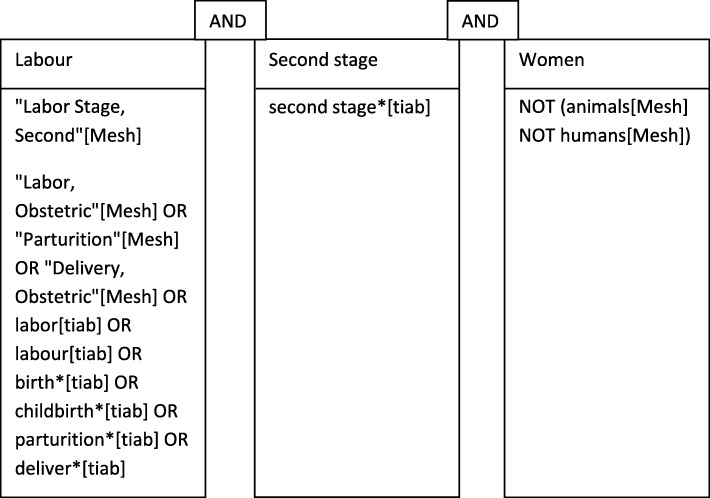
Search terms

We will undertake a systematic literature search based on the Preferred Reporting Items for Systematic Reviews and Meta-Analysis (PRISMA) statement (www.prisma-statement.org) (see Additional file 1). The Peer Review of Electronic Search Strategies (PRESS) 2015 Guideline Statement will be used to enhance the quality and comprehensiveness of the electronic literature search [11]. We will use the PICO framework for quantitative research—P: women in second stage of labour, I: intrapartum intervention by midwives, C: standard care, O: spontaneous physiological birth. For qualitative research, we will use PEO framework—P: women in second stage of labour, E: midwives practices in the second stage of labour, O: spontaneous physiological birth. Systematic searches will identify all pertinent publications, in relevant bibliographic databases: PubMed, EMBASE.com, CINAHL (via Ebsco), PsycINFO (via Ebsco) and The Cochrane Library (via Wiley) from inception, i.e. no publication date restrictions will be applied. An additional search will be performed in the Maternity and Infant Care Database (through MIDIRS). The search strategy will include the Boolean terms OR and AND, the search terms will include controlled terms (for example, MeSH terms in PubMed and Emtree in Embase) as well as free text terms and truncations (*). We will use free text terms only in The Cochrane Library and synonyms and variations of the keywords in all databases (see Fig. 1). The search terms include the following: Labor, Obstetric"[Mesh] OR "Parturition"[Mesh] OR "Delivery, Obstetric"[Mesh] OR labor [tiab] OR labour [tiab] OR birth*[tiab] OR childbirth*[tiab] OR parturition*[tiab] OR deliver*[tiab] Labor, Stage, Second"[Mesh]. All languages will be accepted, as the COST Action network for this study includes individuals who can translate most languages. Animal studies will be excluded. This protocol is registered in the International Prospective Register of Systematic Reviews (PROSPERO; Registration CRD42018088300).

### Study identification and selection

#### Criteria for considering studies for review

#### Inclusion criteria

All studies describing midwives’ care or practice during second stage of physiological birth or normal birth will be included. Both relevant quantitative and qualitative studies will be eligible for review.

#### Exclusion criteria

Studies examining midwifery practice of women that focused only on care during the first or third stage of labour are not eligible. Those studies which include women with an epidural, spinal or instrumental/operative vaginal birth or caesarean section birth will not be included. Furthermore, studies that include women who have not reached full-term pregnancy, have had their pregnancy induced or labour augmented with intravenous oxytocin will not be eligible.

**Fig. 2:**
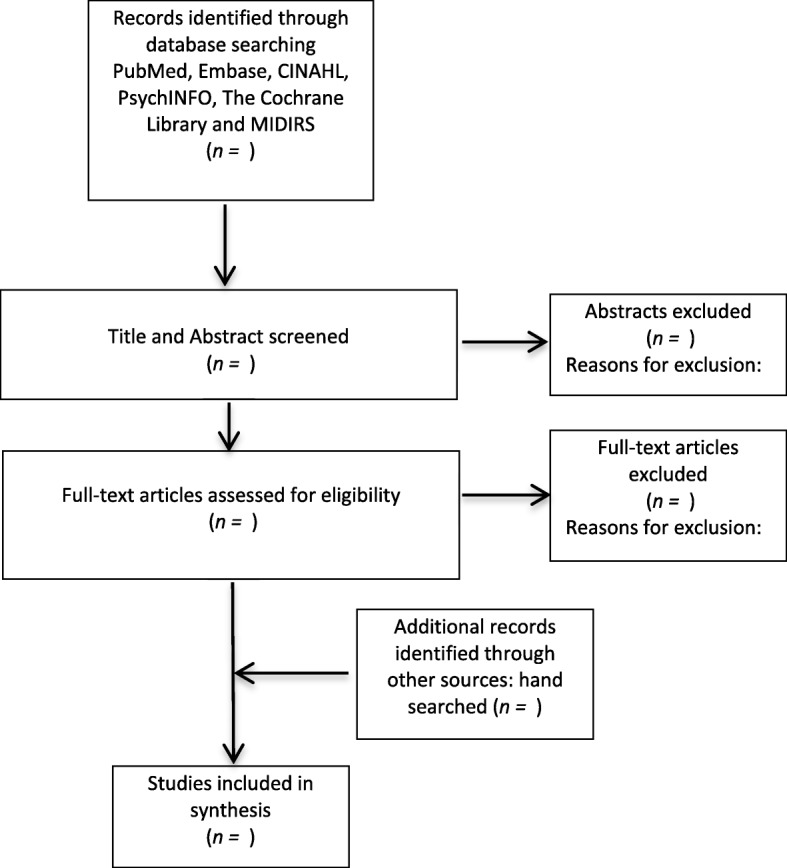
Flow chart

Studies will be selected for inclusion following a two-stage process using *Covidence*. Covidence is a web-based software platform that streamlines the production of systematic reviews, including Cochrane reviews. All available studies, irrespective of language will be included to decrease bias [6]. Within the first stage, each study will have its title and abstract screened by pairs of two independent reviewers (CV, DS, VN, MH). Studies will be excluded if both reviewers consider that a study does not meet eligibility criteria. Full-text manuscripts of all citations that are likely to meet the selection criteria will be retrieved. The final inclusion or exclusion decisions will be made on examination of the full-text manuscripts. Two reviewers will then independently select the studies, which meet the predefined criteria. All disagreements will be discussed and resolved by a senior review author (CV or MH). We will report the reasons for exclusion for each study, and a flow chart (Fig. 2) will be used to present the process of screening and inclusion of the studies in this review. Articles that pass the two-stage screening process will then be assessed for risk of bias and have their reference lists hand searched.

### Study quality assessment

Study quality assessment will be performed by two reviewers independently. The tool utilised to assess the quality of evidence will depend on each study’s methodological approach. To assess the risk of bias in randomised controlled trials, the Cochrane Collaboration’s tool for assessing risk of bias will be used [7]. For all other study types, the Critical Appraisal Skills Programme (CASP) criteria will be used [3]. The Grading of Recommendations Assessment, Development and Evaluation (GRADE), Cochrane’s recommended approach for grading the body of evidence, will be used for quantitative studies. Confidence in the Evidence from Reviews of Qualitative research (CERQual) will be used for grading the confidence in the evidence of qualitative studies.

### Analysis

Depending on the findings, a meta-analysis and/or a meta-synthesis will be undertaken.

## Discussion

A midwife’s practice can be influenced by education and cultural practices, but ultimately it should be informed by up-to-date research evidence. This systematic review will comprehensively collate, analyse and synthesise the available evidence relating to what midwives do to facilitate physiological birth. This will help to formulate midwifery practice, education and future research recommendations that support high-quality intrapartum care during the second stage of labour.

## Additional file

Additional file 1: The PRISMA-P 2015 Checklist (PDF 373 kb)

## Abbreviations

CERQual: Confidence in the Evidence from Reviews of Qualitative research
GRADE: Grading of Recommendations Assessment, Development and Evaluation
PEO: Population, Exposure, Outcomes
PICO: Patient or Population, Intervention, Comparison, Outcome
PRISMA: Preferred Reporting Items for Systematic Reviews and Meta-Analysis
PROSPERO: International Prospective Register of Systematic Reviews
WHO: World Health Organization
